# An *in silico* study on molecular level interactions of host Siderocalin with siderophores from *Mycobacterium tuberculosis* and other bacterial species

**DOI:** 10.1186/1471-2334-14-S3-O15

**Published:** 2014-05-27

**Authors:** Tanu Goyal, Sharmila Anishetty

**Affiliations:** 1 Centre for Biotechnology, Anna University, Chennai, India

## Background

Tuberculosis (TB) drug research and development has witnessed resurgence in recent times primarily due to the high mortality rates despite the availability of front line drugs and a prominent BCG vaccine. Synergy of TB with HIV is another factor which has warranted a search for newer therapeutic interventions. Treatment of latent TB infection is also the need of the hour. Iron acquisition is an important virulence mechanism of *Mycobacterium tuberculosis* (MTB). To scavenge iron, bacterial species have developed high affinity, low molecular weight iron chelators termed as siderophores. To counteract the detrimental effect of microbial iron acquisition systems, the host secretes a 21kDa lipocalin protein (Siderocalin) that binds with these iron laden siderophores in an attempt to restrict the growth of MTB within host macrophages.

## Methods

In the current study, using molecular docking tools, we assessed the interactions of host Siderocalin with Mycobactin, Parabactin and Cepabactin structurally non similar siderophores from three different bacterial species-MTB, Paracoccus and *Burkholderia cepacia*. A comparison of molecular level interactions of Siderocalin with these siderophores was performed.

## Results

Siderocalin forms energetically favourable and stable complexes with parabactin and cepabactin. However the unfavourable positive binding energies for mycobactin- a salicylate derived MTB siderophore indicated that siderocalin does not dock well with mycobactin.

## Conclusion

Siderocalin probably fails to disrupt the role of mycobactin in acquiring iron for mycobacteria thereby helping the pathogen to survive leading to progression of disease.

